# KEGG2Net: Deducing gene interaction networks and acyclic graphs from KEGG pathways

**DOI:** 10.14806/ej.26.0.949

**Published:** 2021-03-05

**Authors:** Sree K. Chanumolu, Mustafa Albahrani, Handan Can, Hasan H. Otu

**Affiliations:** 1 Department of Electrical and Computer Engineering, University of Nebraska-Lincoln, Lincoln, NE, United States

## Abstract

The Kyoto Encyclopedia of Genes and Genomes (KEGG) pathway database provides a manual curation of biological pathways that involve genes (or gene products), metabolites, chemical compounds, maps, and other entries. However, most applications and datasets involved in omics are gene or protein-centric requiring pathway representations that include direct and indirect interactions only between genes. Furthermore, special methodologies, such as Bayesian networks require acyclic representations of graphs. We developed KEGG2Net, a web resource that generates a network involving only the genes represented on a KEGG pathway with all of the direct and indirect gene-gene interactions deduced from the pathway. KEGG2Net offers four different methods to remove cycles from the resulting gene interaction network, converting them into directed acyclic graphs (DAGs). We generated synthetic gene expression data using the gene interaction networks deduced from the KEGG pathways and performed a comparative analysis of different cycle removal methods by testing the fitness of their DAGs to the data and by the number of edges they eliminate. Our results indicate that an ensemble method for cycle removal performs as the best approach to convert the gene interaction networks into DAGs. Resulting gene interaction networks and DAGs are represented in multiple user-friendly formats that can be used in other applications, and as images for quick and easy visualisation. The KEGG2Net web portal converts KEGG maps for any organism into gene-gene interaction networks and corresponding DAGS representing all of the direct and indirect interactions among the genes.

## Introduction

The KEGG pathway database provides hundreds of manually curated maps that involve molecular interactions between gene products, compounds, maps, DNA, RNA, and other molecules (Kanehisa and Goto, 2000). The maps are categorised under seven groups, such as “Metabolism” or “Environmental Information Processing,” which are stored in proprietary files in XML format, called KGML. There exist numerous approaches that process the KEGG pathway maps, such as KEGGtranslator (Wrzodek *et al.*, 2011), KEGGParser (Arakelyan and Nersisyan, 2013), CyKEGGParser (Nersisyan *et al.*, 2014), KEGGgraph (Zhang and Wiemann, 2009), KEGGconverter (Moutselos *et al.*, 2009), and graphite (Sales *et al.*, 2012) among others (Wrzodek *et al.*, 2013). These approaches convert KGML files to other formats (*e.g.*, SBML, BioPAX) to be used in applications for data visualisation (*e.g.*, Cytoscape) or graph-theoretic analysis (*e.g.*, MATLAB®, Bioconductor).

Although these approaches are extremely useful, they do not provide a representation that only includes genes deduced from the KEGG pathways considering all of the direct and indirect gene interactions. However, when analysing experimental datasets that only involve the genes (or gene products) in the context of KEGG pathways (*e.g.*, transcriptomic or proteomic data), an interaction network that only involves these molecules is required. Among the existing tools, KEGGgraph provides a *“genesOnly”* parameter that results in a gene-oriented graph; but that approach only deduces the direct gene interactions provided in the maps. graphite also represents gene-only networks, but it does not take into account all of the compounds between the genes to obtain the exhaustive set of indirect interactions – some compounds based on their identity or localisation are ignored. Indeed, there is a need for an approach that recovers all of the direct and indirect gene-gene interactions from a KEGG pathway that can be used in downstream analysis involving data coming only from these molecules.

KEGG pathways include cycles that may be problematic in analysis approaches, such as Bayesian networks (BNs), which use directed acyclic graphs (DAGs) (Friedman *et al.*, 2000; Isci *et al.*, 2014; Isci *et al.*, 2011; Korucuoglu *et al.*, 2014; Liu *et al.*, 2016). None of the existing approaches that process KEGG pathways provide DAGs as their output. Furthermore, there has been no study that compares different cycle removal methods in the context of the fitness of biological data to the resulting DAGs.

In light of these observations and perceived needs, we developed KEGG2Net, a web resource that converts KEGG pathways into gene interaction networks involving all of the direct and indirect relations between the genes that can be deduced from the map. KEGG2Net offers four alternative methods to convert the resulting gene interaction networks into DAGs. This paper also provides a comparative assessment of the cycle removal methods via their fitness to the data obtained from the original gene interaction network deduced from KEGG.

## Implementation

Given the KGML file for a pathway map, the engine parses the file to obtain an adjacency matrix that represents all of the interactions (relation, reaction, *etc.*) between all of the node types (compound, map, gene, *etc.*) defined in the file. In this graph, if there exists a path between two genes that contain non-gene nodes only, then an indirect relation between the two genes is established by placing an edge between them. Next, nodes that are not genes are removed from the adjacency matrix. This way, the resulting graph represents all of the direct and indirect interactions between the genes that can be deduced from the KEGG pathway map.

The resulting gene network may contain cycles that are removed using four different methods: a depth-first search (DFS) (Suominen and Mader, 2014), a greedy local heuristic to the minimum feedback arc set (MFAS) problem (Eades *et al.*, 1993), and two graph-hierarchy-based methods where the hierarchy is inferred either through PageRank (PR) (Page *et al.*, 1999) or an ensemble method (EN) (Sun *et al.*, 2017) based on the TrueSkillTM (Herbrich *et al.*, 2007) and social agony (Gupte *et al.*, 2011) metrics. The DFS-based approaches use fast, simple heuristics to remove back edges, MAFS-based approaches try to minimise the number of edges removed, and hierarchy-based methods define a hierarchy in the graph first and then devise an edge removal strategy that prioritises the maintenance of the defined hierarchy as much as possible.

The input to KEGG2Net is the KGML files for the pathways that belong to the organism selected by the user. The output of KEGG2Net consists of the gene interaction networks deduced from the pathways and four DAGs per network where the cycles are removed by the aforementioned four algorithms. The networks and DAGs are represented as adjacency matrices and simple interaction files (a.k.a. SIF or .sif format) for use in the downstream analysis by other software and for visualisation purposes.

**Figure 1.**
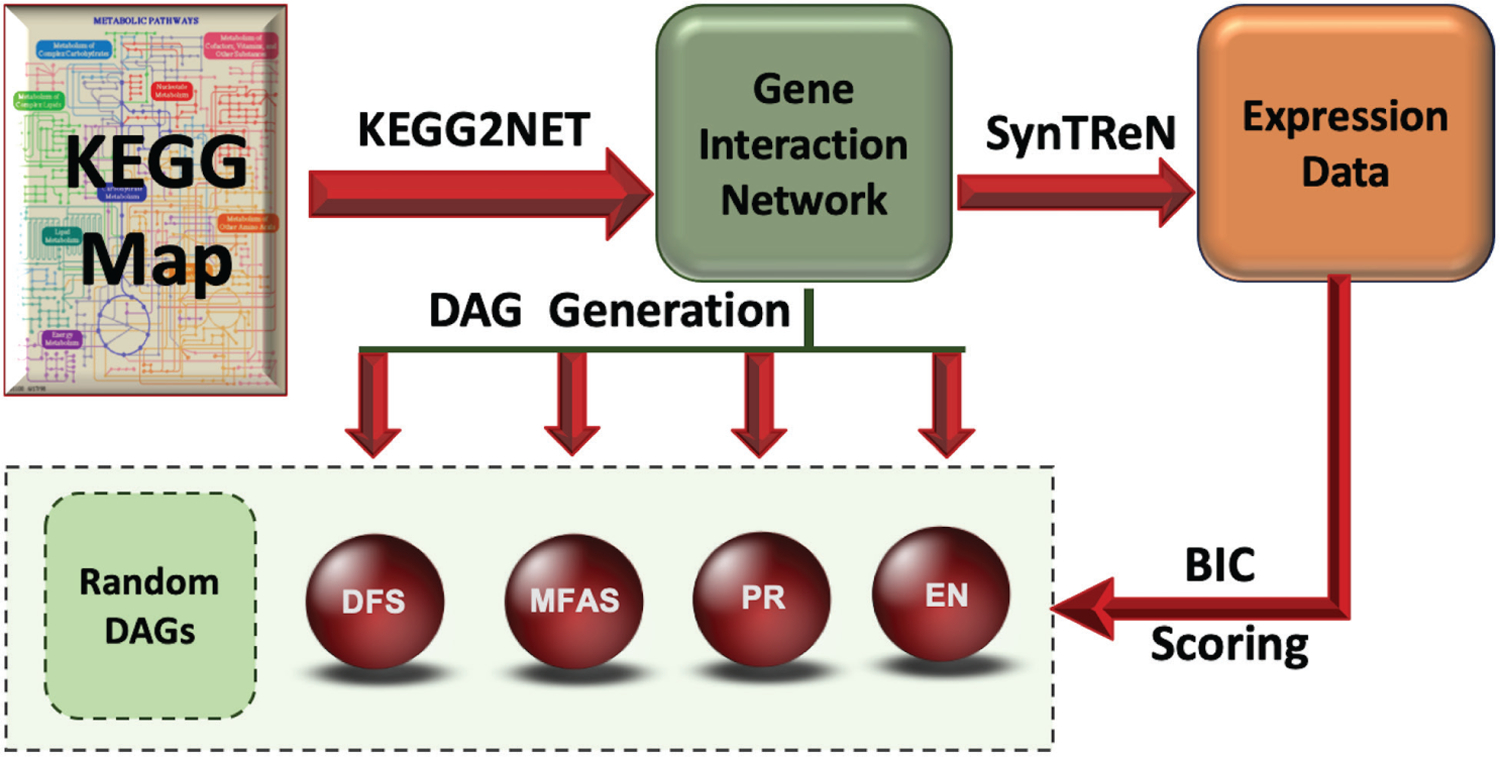
KEGG2Net workflow and directed acyclic graph (DAG) generation. Each KEGG pathway is converted into a gene interaction network where only the genes in the pathway are represented, and all direct and indirect interactions among the genes are preserved. For each network, four DAGs (using four alternative cycle removal methods depth-first search (DFS), minimum feedback arc set (MFAS), PageRank (PR), and ensemble (EN)) and 1,000 random DAGs for each of the four DAGs are generated. The random DAGs follow the node and edge statistics of their corresponding DAG. For each gene interaction network, synthetic gene expression data is generated using SynTRen and the fitness of the four DAGs (and the corresponding 4×1,000 = 4,000 random DAGs) is assessed using Bayesian Information Criterion (BIC) scoring.

In order to compare the accuracy of different cycle removal methods and to provide a sample output using graph images for visualisation purposes, we applied the KEGG2Net approach on the 335 available human KEGG pathways. The KEGG2Net workflow adopted in this paper is shown in Figure 1.

Out of the 335 pathways, we considered only the networks with six or more non-isolated nodes, which left us with 280 networks. Of these networks, 150 did not have any cycles. For each of the remaining 130 that were cyclic, a synthetic gene expression data fitting the graph topology was generated using SynTReN (v. 1.2) (Van den Bulcke *et al.*, 2006). The expression data was processed to be used by BN scoring methods as previously described (Isci *et al.*, 2011). The four cycle removal methods were applied to the 130 networks with cycles generating four DAGs per network, which were scored based on the Bayesian Information Criterion (BIC) in the *bnlearn* R package (Scutari, 2010) using the processed synthetic expression data. For each of the four DAGs per network, we generated 1,000 random DAGs (with the same number of edges and nodes as the original DAG) and obtained their BIC scores based on the same processed synthetic expression data to assess the goodness of the original DAG’s score.

## Results

Our web portal provides the gene interaction networks obtained for all of the 335 human KEGG pathways. For the networks that have cycles, we also list the DAGs obtained using the four methods. The gene interaction networks and the DAGs obtained from them are represented by adjacency matrices, SIF format files, and graph images. KEGG2Net can be used for all of the organisms listed in KEGG where the user can download the relevant network and DAG files via our web portal.

One direct way to assess the performance of different cycle removal methods is to compare the number of edges removed by each method. However, this approach does not infer the degree to which the topology and the dependency structure among the nodes of the network are preserved in the DAGs. For this purpose, we first generated synthetic gene expression data that follow the regulatory dynamics explained by the gene interaction network deduced by KEGG2Net. We then scored each of the four DAGs with this expression data using BIC scoring, where a higher score indicated a better fit. Finally, for each of the four DAGs that result from the given network, we generated 1,000 random DAGs (i.e., 4,000 random DAGs per network) where the random DAGs had the same number of nodes and links as the DAG they were associated with. This exercise was repeated for all of the 130 networks, and the DAG statistics were compared.

The complete set of results for our simulations are given in the Supplementary Data, which involves the 130 KEGG2Net gene interaction networks with six or more non-isolated nodes and a cycle. We provide the original pathway’s ID, name, number of edges, number of nodes, the number of edges removed by the four cycle removal methods, the rank of each method for each pathway based on the edges removed, and the rank of the score for each method (among themselves and their 1,000 random DAGs).

**Figure 2.**
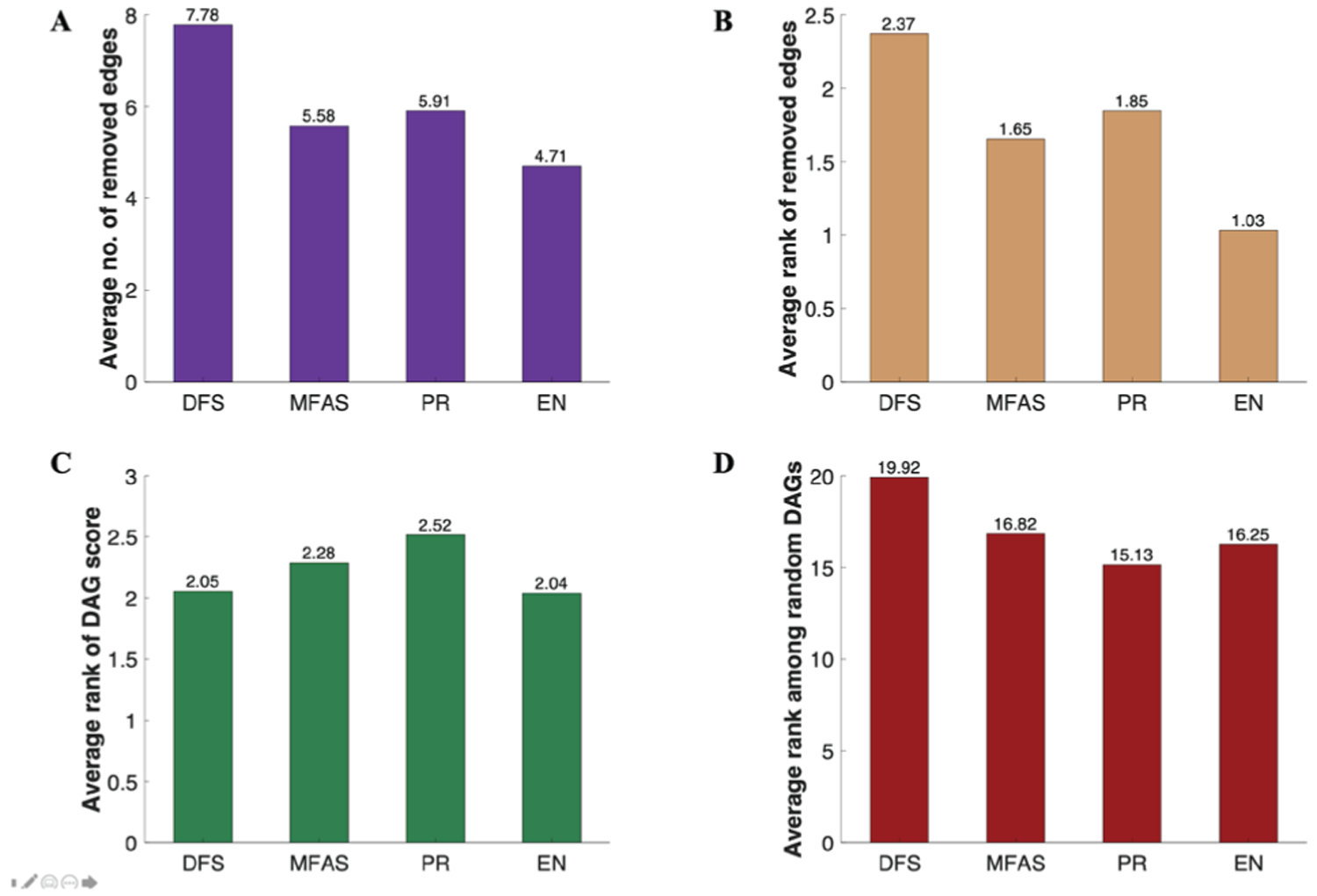
Directed acyclic graph (DAG) statistics. Based on the four cycle removal methods, depth-first search (DFS), minimum feedback arc set (MFAS), PageRank (PR), and ensemble (EN), applied on 130 gene interaction networks deduced from pathways using KEGG2Net, **A** average number of edges removed; **B** average rank of the method (per network) based on the number of edges removed (ascending); **C** average rank of the method’s DAG score (per network) among the four methods (descending); **D** average rank of the method’s DAG score (per network) among the 1,000 random DAGs that has the same numbers of edges and nodes as the DAG (descending). For parts **C** and **D**, Bayesian Information Criterion (BIC) scoring is used to assess the fitness of the DAGs to the synthetic data generated based on the gene interaction network (obtained by KEGG2Net).

These results, summarised in Figure 2, showed that on average, the EN method required the least number of edges to be removed and provided the DAG with the highest BIC score.

On average, the EN method removed 4.71 edges per pathway; and it was the method that required the least number of edges to be removed in 127 out of 130 networks. The EN method accomplished an average rank of 1.03 in all networks, where 1 represented the rank that removed the minimum number of edges.

The average rank of the EN score among the four DAGs was also the highest, where it attained an average rank of 2.04. In other words, on average, the EN DAG provided the best topology that fit the synthetic expression data. The only category where the EN method was outperformed was its average rank among the 1,000 random DAGs. On average, 16.25 random DAGs for a given network performed better than the EN DAG, whereas this number was 15.13 for the PR DAG, the only method that beat the EN approach in this category. Given the comprehensive evaluation summarised in Figure 2, we recommend EN as the method of choice for DAG generation.

## Discussion

In this work, we provide a web resource that converts KEGG pathways into gene interaction networks representing all of the direct and indirect interactions between the genes. We also provide four alternative ways of converting the resulting graph into a DAG. Our results showed that the EN method finds the best DAG that explains the underlying hierarchy and dependency structure defined in the interaction network with the minimum number of edge removals. Our web portal lists the graph structure and the four DAGs for each of the KEGG human pathways. The KEGG2Net web resource can be used to obtain the networks and DAGs for any organism listed in the KEGG database.

## Supplementary Material

supplementary material

## Key Points

- Deduces only the gene-gene interactions from a KEGG pathway considering all direct and indirect interactions.
- Provides networks that are ready to be applied to transcriptomic or proteomic data for system-level analysis.
- Using multiple alternative methods, converts networks to directed acyclic graphs (DAGs) that can be used in methodologies such as Bayesian networks, which require DAGs.
- Generates multiple output formats that can be directly used in different network visualisation or analysis software.
- Reports a comparative analysis of cycle removal methods for biological pathways.
